# How Does Severe Acute Respiratory Syndrome-Coronavirus-2 Affect the Brain and Its Implications for the Vaccines Currently in Use

**DOI:** 10.3390/vaccines10010001

**Published:** 2021-12-21

**Authors:** Philip R. Oldfield, Jennifer Hibberd, Byram W. Bridle

**Affiliations:** 1 Scientific and Regulatory Consultant, Rigaud, QC J0P 1P0, Canada; philip.oldfield@poldfieldbc.com; 2 Faculty of Dentistry, University of Toronto, Toronto, ON M5G 1X3, Canada; drjenniferhibberd@gmail.com; 3 Department of Pathobiology, University of Guelph, Guelph, ON N1G 2W1, Canada

**Keywords:** severe acute respiratory syndrome-coronavirus-2 (SARS-CoV-2), novel coronavirus disease that emerged in 2019 (COVID-19), cytokines, spike protein, blood–brain barrier (BBB), angiotensin-converting enzyme 2 (ACE2), Parkinson’s disease, Lewy body dementia, good laboratory practice

## Abstract

This mini-review focuses on the mechanisms of how severe acute respiratory syndrome-coronavirus-2 (SARS-CoV-2) affects the brain, with an emphasis on the role of the spike protein in patients with neurological symptoms. Following infection, patients with a history of neurological complications may be at a higher risk of developing long-term neurological conditions associated with the α-synuclein prion, such as Parkinson’s disease and Lewy body dementia. Compelling evidence has been published to indicate that the spike protein, which is derived from SARS-CoV-2 and generated from the vaccines currently being employed, is not only able to cross the blood–brain barrier but may cause inflammation and/or blood clots in the brain. Consequently, should vaccine-induced expression of spike proteins not be limited to the site of injection and draining lymph nodes there is the potential of long-term implications following inoculation that may be identical to that of patients exhibiting neurological complications after being infected with SARS-CoV-2. However, further studies are needed before definitive conclusions can be made.

## 1. Introduction

Severe acute respiratory syndrome-coronavirus-2 (SARS-CoV-2) is the causative agent of the novel coronavirus disease that emerged in 2019 (COVID-19). It prompted the declaration of a global pandemic that has persisted for more than a year. According to the website “Worldometer” (https://www.worldometers.info/coronavirus/ accessed on 14 December 2021), there were 271.7 million cases, 5.3 million deaths, and 244.3 million recoveries from COVID-19. COVID-19 is primarily being considered a respiratory disease. However, unlike the common flu, other organs, including the brain, are also severely affected in some patients [1]. Evidence shows that SARS-CoV-2 has the ability to affect the brain directly, with a new set of symptoms: loss of smell and taste, severe headaches, debilitating fatigue, trouble thinking clearly (brain fog), seizures, strokes, and various degrees of paralysis [2]. Numerous reliable studies have already been published on this topic and are referenced in this article. Results from preclinical studies and several revealing patient case histories will be used to explain the mechanisms involved.

## 2. Lessons Learnt from Similar Coronaviruses

**Figure 1:**
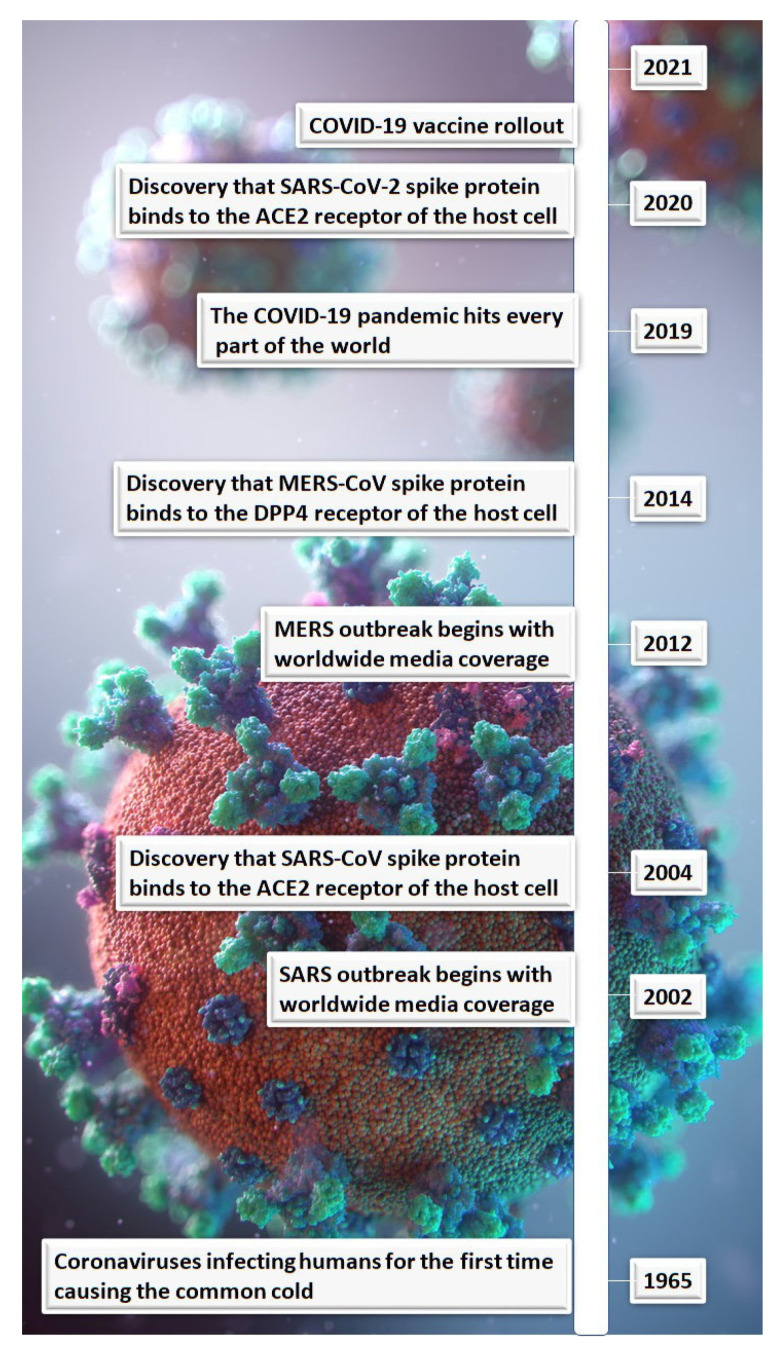
Timeline of coronaviruses and spike protein receptor targets.

The first coronavirus to affect humans was the common cold in 1965. Since then, there was the SARS-CoV (the virus that causes SARS) outbreak in 2002, followed by the Middle East respiratory syndrome (MERS)-CoV (the virus that causes MERS) outbreak ten years later (Figure 1). At the time, these outbreaks caused worldwide concern with case fatality rates of 9.5% and 34.4%, respectively [3]. Although the case fatality rates for SARS and MERS were higher than that for COVID-19, which is 2.3% [3], outbreaks of the former two infectious agents were contained (Table 1). So, what do these previous infections have in common with the SARS-CoV-2 virus?

MERS-CoV binds to the dipeptidyl peptidase 4 receptor (also known as CD26). What made MERS so deadly was the fact that the receptor to which the virus bound was involved in modulating the host’s immune system. T-cells were inhibited from binding to the CD26 receptor, resulting in a delayed adaptive immune response. While MERS-CoV bound to the CD26 receptors on macrophages, which forced the body to maintain its innate immune response, this resulted in the patient having a fever, initiation of a severe inflammatory reaction, and death of cells in the immune system that expressed CD26, thus making the condition worse [8,9] and giving rise to a poor prognosis (Table 1).

Both SARS-CoV and SARS-CoV-2 target the same receptor, angiotensin-converting enzyme 2 (ACE2), on host cells, and this is probably the reason why the clinical symptoms are similar (Table 1). With SARS and, to a lesser extent, with COVID-19, patients can feel well one moment and then, over a short period of time, develop breathing problems requiring oxygen and hospitalization. To gain insight into what might be happening in these patients, one needs to consider the role of the angiotensin-rennin system that regulates blood pressure and fluid balance. When the spike protein of SARS-CoV-2 binds the ACE2 receptor, the body control mechanism for blood pressure and fluid balance is disrupted, causing a buildup of fluid in the lungs and leading to pneumonia and respiratory failure [10]. This exemplifies not only the virus causing the body to work against itself but the difficulty facing physicians when it comes to effective treatment. The angiotensin-rennin system doesn’t have redundancy. Therefore, once this the system becomes dysregulated, the accumulation of fluid in the lungs is inevitable [10]. This being the case, it can be anticipated that such patients can experience rapid declines in respiratory function, with difficulty breathing resulting in low oxygen saturation, permanent lung damage, and possibly other organ damage due to hypoxia, made even worse by mechanical ventilation [11]. Long-term effects of SARS-CoV and MERS-CoV are known to be associated with respiratory impairment due to permanent lung damage and psychological issues such as depression directly affecting a person’s daily life years following recovery from the initial infection. A proportion of these patients are unable to return to full-time employment [12,13]. Since SARS-CoV-2 targets the same receptor as SARS-CoV, a follow-up of seriously ill hospitalized patients is recommended.

Therefore, it can be concluded that the spike protein of the virus plays a key role in the pathogenesis of these diseases. These examples also illustrate the need to determine the molecular basis of disease to facilitate effective treatments.

## 3. SARS-CoV-2 and Neurological Symptoms

Although rare, many of the serious neurological symptoms associated with COVID-19 are due to hypoxia, cytokine storms, and blood clots, all of which contribute to damaging neurons in the brain. Loss of smell and taste (anosmia), severe headaches, debilitating fatigue, trouble thinking clearly (brain fog), seizures, strokes, and various degrees of paralysis are some of these symptoms [2].

Often, one of the first symptoms of COVID-19 is the loss of smell and taste. With the olfactory nerve anatomically close to the brain, this nerve pathway would be the ideal means for SARS-CoV-2 to enter the brain in the early stages of the disease [14]. However, it remains unclear as to whether SARS-CoV-2 can spread into the brain via this route. It has been reported that the sustentacular and stem cells in the olfactory epithelium express ACE2 and are vulnerable to being infected by SARS-CoV-2. Conversely, the olfactory sensory neurons do not express ACE2, suggesting SARS-CoV-2 cannot gain access to the brain [15,16] (not yet peer reviewed). It was, therefore, concluded that damage to the olfactory epithelium underlies clinical anosmia, rather than neuronal injury.

Nonetheless, there is an alternative route by which SARS-CoV-2 can enter the brain as the disease progresses [14]. The spike proteins of SARS-CoV-2 bind to the ACE2 receptors expressed by the endothelial cells of the brain’s vasculature, facilitating entry into these cells, enabled by the enzyme transmembrane protease serine 2. The viral damage to the endothelial cells initiates an inflammatory reaction that is essentially a cytokine storm, activating neutrophils and macrophages in the blood as well as microglia and astrocytes in the brain. Endothelial damage and the direct activation of platelets by SARS-CoV-2 via spike protein/ACE2 interactions [17] can also promote the formation of micro-thrombi, which in turn can develop into blood clots. Thus, with this destabilization of the blood–brain barrier (BBB) caused by the cytokines in conjunction with the chemokine monocyte chemoattractant protein 1, SARS-CoV-2 can then freely pass through the BBB.

Once in the brain, the virus would then have the opportunity to replicate. SARS-CoV-2 has the ability to replicate using the neuron cell machinery as demonstrated using human brain cell organoids [18]. In addition, brain biopsies from patients that had been diagnosed with COVID-19 showed that SARS-CoV-2 was able to target and replicate in the neurons themselves [18]. However, it became apparent that neuroinflammation resulting in a cytokine storm was a major factor resulting in the activation of microglia. This causes an increase in kynurenine, quinolinic acid, and glutamate and a corresponding depletion of the neurotransmitters serotonin, dopamine, and norepinephrine, which is responsible for the neuropsychiatric symptoms associated with COVID-19 and neuron damage [14].

## 4. COVID-19 and a Possible Connection with Parkinson’s Disease

Based upon other viral infections affecting the brain, it has been suggested that SARS-CoV-2 infection of the central nervous system may lead to long-term neurological consequences for some patients [19]. A pre-print entitled “SARS-CoV-2 causes brain inflammation and induces Lewy body formation in macaques” [20] (not yet peer reviewed) emphasizes this concern. In this study, the treatment group, four male rhesus and four male cynomolgus monkeys, were experimentally infected with SARS-CoV-2. The control group was comprised of two male rhesus and two male cynomolgus monkeys. The monkeys experienced asymptomatic infections and were euthanized between five to six weeks post-infection, and their brain tissues were stained for Lewy bodies by immunohistochemistry. Formation of intracellular Lewy bodies was observed in the midbrain region of the caudate nucleus of all eight of the infected monkeys, while Lewy bodies were absent in the brains of all monkeys in the control group. These results provided compelling evidence that SARS-CoV-2 has the ability to initiate α-synuclein misfolding and is, therefore, responsible for Lewy body formation in SARS-CoV-2-infected monkeys.

Should the same misfolding of α-synuclein (i.e, α-synuclein prion), its aggregation, and the formation of Lewy bodies occur in the brains of patients who had previously recovered from SARS-CoV-2, this could potentially lead to neurodegenerative diseases, such as Parkinson’s disease and Lewy body dementia, years, if not decades, later. It is, therefore, essential to provide follow-up for patients diagnosed with COVID-19 who exhibit neurological complications post-infection [21].

## 5. Pathology Attributed to the SARS-CoV-2 Spike Protein

The vaccines that have currently received authorization to reduce the severity of COVID-19 employ either mRNA or DNA to transfect human cells to produce the spike protein of SARS-CoV-2, which then becomes the antigen to be targeted by the body’s immune system.

An indication that the spike protein has the potential to cross the BBB was evident from an in vitro study [22] suggesting that purified spike proteins from SARS-CoV-2 can initiate the pro-inflammatory response in endothelial cells in the brain, thereby destabilizing the BBB. Secondly, an in vivo study in mice demonstrated that the S1 subunit of the spike protein readily crossed the BBB, entering the brain parenchyma, after the iodinated S1 subunit was administered intravenously to male mice [23]. Connecting these phenomena to the vaccine administration site, which is the deltoid muscle, was the observation that the spike protein S1 subunit was detectable in the systemic circulation up to approximately two weeks post-immunization in eleven out of thirteen healthcare workers [24]. Although concentrations of the S1 subunit were low, this study provides proof-of-principle that spike proteins or components thereof can get into circulation following inoculation. Considering the relatively low incidence of severe adverse events caused by COVID-19 vaccines, if circulating spike proteins were drivers of any side effects the sample size of this study was far too small to identify potentially rare individuals with relatively high concentrations of circulating spike proteins. Further, it is possible that spike proteins were already bound to ACE2 receptors on endothelial cells.

It was demonstrated that the spike protein S1 subunit alone is responsible for initiating pro-inflammatory responses via Toll-like receptor 4-mediated signaling [25]. In addition, the spike protein alone, when bound to ACE2 on the surface of platelets, can regulate platelet function, which in turn results in blood clot formation [17]. Thus, it is evident that the spike protein on its own can recapitulate key aspects of the pathogenesis observed following infection with SARS-CoV-2.

## 6. What Are the Implications for the COVID-19 Vaccines Currently Being Used?

During the pandemic, vaccine manufacturers focused mainly on assessing the efficacy of their products [26,27,28]. However, following experience with other coronaviruses as mentioned earlier, it was already known that the spike proteins are largely responsible for the observed pathology. Therefore, a theoretical scenario exists where a certain proportion of people receiving the COVID-19 vaccine, likely from among those with neurological side effects (e.g., severe headaches), may exhibit neurological symptoms of synucleinopathies diagnosed as Parkinson’s disease and/or Lewy body dementia up to two to three decades post-immunization. In the U.S. Food and Drug Administration’s (FDA) briefing document, Pfizer stated the following with respect to their COVID-19 vaccine: “Following authorization of the vaccine, use in large numbers of individuals may reveal additional, potentially less frequent, and/or more serious adverse events not detected in the trial safety population of nearly 44,000 participants over the period of follow-up at this time. Active and passive safety surveillance will continue during the pos- authorization period to detect new safety signals” [26]. The other manufacturers made similar statements. Obviously, the pharmaceutical industry has taken it upon itself to initiate active and passive safety surveillance during this post-authorization period. On 23 August 2021, BioNTech Manufacturing GmbH (in partnership with Pfizer Inc., New York, NY, USA) received US FDA’s Biologics License Application authorization (STN: BL 125742/0) for their COVID-19 mRNA vaccine BNT162b2, now marketed as “COMIRNATY” [29].

## 7. Discussion

Few would have thought, when the COVID-19 pandemic was declared in early 2020 and labeled as a respiratory illness, that discussion would progress to the question of serious neurological consequences. We have already recognized the need to closely monitor and screen for α-synuclein prions in patients who have experienced neurological complications, but what about people who have been vaccinated? Over this past year, there have been several publications indicating that the neurological side effects of the COVID-19 vaccines are extremely rare. This is true for the more serious adverse events as stated by the authors, considering the number of actual doses administered [30,31,32,33]. However, that does not detract from the fact that these individuals need to be followed up. In addition, it is worth noting that according to the label [34] of the recently FDA-approved COVID-19 vaccine “COMIRNATY”, submitted by BioNTech Manufacturing GmbH (in partnership with Pfizer Inc.), the occurrence of acute neurological symptoms (i.e., fatigue and/or headaches), although not considered to be serious, were high, especially in the younger age group (16 through to 55 years of age). Such neurological symptoms, especially if prolonged, could well be indicative of inflammation, which can act as the trigger giving rise to synucleinopathies such as Parkinson’s disease up to two to three decades post-immunization [21]. Therefore, individuals who have experienced adverse neurological effects after being administered a COVID-19 vaccine should also be followed up.

Of interest is the mode of action of the COVID-19 vaccines currently used, where the end product is the SARS-CoV-2 spike protein, the intended target being the antigen-presenting cells of the immune system. However, there are indications that the spike protein generated by these vaccines may have off-target effects [17,34,35,36]. There is no evidence that distribution and/or toxicokinetic studies of the spike protein were performed [34,37]. With this in mind, it would be prudent to follow up subjects who experience neurological side effects as a result of the COVID-19 vaccines, in addition to the hospitalized patients with COVID-19 who had neurological complications. With the COVID-19 vaccines, an assumption was made that the spike protein produced in the host cells would not be shed into the systemic circulation. Hence, additional data are required to determine the toxicity and distribution of the spike protein, which is already known to cause disease. Perhaps a standard 28-day study with good laboratory practice could be initiated that not only mimics the concentration that enters the plasma post-vaccination [24] but also exceeds it, in order to provide a good safety margin in a suitable species. Other regulated studies may also be deemed appropriate to fill the knowledge gap. The Guidance for Industry that perhaps should have been used is the Preclinical Assessment of Investigational Cellular and Gene Therapy Products [38], although, like the Development and Licensure of Vaccines to Prevent COVID-19 Guidance for Industry [39], in which the recommendations were also non-binding, the former guidance does recommend more extensive non-clinical studies—including full histopathology—and in the introduction states: “The Center for Biologics Evaluation and Research (CBER)/Office of Cellular, Tissue, and Gene Therapies (OCTGT) is issuing this guidance to provide sponsors and individuals that design and implement preclinical studies with recommendations on the substance and scope of preclinical information needed to support clinical trials for investigational cellular therapies, gene therapies, therapeutic vaccines, xenotransplantation, and certain biologic–device combination products which OCTGT reviews (hereinafter referred to as CGT products).”.

Until such definitive studies are carried out and results substantiated, it lends consideration for caution when deciding whether to administer the COVID-19 vaccines to the younger age groups.

## Figures and Tables

**Table 1:** Characteristics of severe acute respiratory syndrome-coronavirus (SARS-CoV), Middle East respiratory syndrome-coronavirus (MERS-CoV), and SARS-CoV-2.

Coronavirus	Host Cell Target Receptor [3]	Clinical Symptoms	Death Rate (%) [3]
SARS-CoV	Angiotensin-converting enzyme 2 (ACE2)	Fever, Tiredness, Chills, Muscle aches, Dry cough, Difficulty breathing, Headaches, Sore throat, Diarrhea [4]	9.5
MERS-CoV	Dipeptidyl peptidase 4	Fever, Cough, Shortness of breath [5]	34.4
SARS-CoV-2	ACE2	Fever, Dry cough, Chills, Difficulty breathing, Tiredness, Body aches, Headaches, Loss of taste or smell, Sore throat, Diarrhea [6,7]	2.3
